# Three novel *FHL1* variants cause a mild phenotype of Emery‐Dreifuss muscular dystrophy

**DOI:** 10.1002/humu.24415

**Published:** 2022-07-16

**Authors:** Josefine d. S. Borch, Thomas Krag, Sonja D. Holm‐Yildiz, Hakan Cetin, Tuva A. Solheim, Freja Fornander, Volker Straub, Morten Duno, John Vissing

**Affiliations:** ^1^ Department of Neurology, Rigshospitalet, Copenhagen Neuromuscular Center University of Copenhagen Copenhagen Denmark; ^2^ Department of Neurology Medical University of Vienna Vienna Austria; ^3^ John Walton Muscular Dystrophy Research Centre, Translational and Clinical Research Institute Newcastle University and Newcastle Hospitals NHS Foundation Trust Newcastle Upon Tyne UK; ^4^ Department of Clinical Genetics, Section 4062, Rigshospitalet University of Copenhagen Copenhagen Denmark

**Keywords:** cardiomyopathy, EDMD6, Emery‐Dreifuss muscular dystrophy, FHL1, muscular dystrophy

## Abstract

Emery‐Dreifuss muscular dystrophy (EDMD) is a hereditary muscle disease, characterized by the clinical triade of early‐onset joint contractures, progressive muscle weakness, and cardiac involvement. Pathogenic variants in *FHL1* can cause a rare X‐linked recessive form of EDMD, type 6. We report three men with novel variants in *FHL1* leading to EDMD6. The onset of muscle symptoms was in late adulthood and muscle weakness was not prominent in either of the patients. All patients had hypertrophic cardiomyopathy and one of them also had cardiac arrhythmias. Western blot performed on muscle biopsies from two of the patients showed no FHL1 protein expression. We predict that the variant in the third patient also leads to the absence of FHL1 protein. Complete loss of all FHL1 isoforms combined with mild muscle involvement supports the hypothesis that loss of all FHL1 isoforms is more benign than the cytotoxic effects of expressed FHL1 protein with pathogenic missense variants.

## Graphical abstract

Three novel variants in FHL1 associated with no FHL1 protein. Complete loss of all FHL1 isoforms leads to mild muscle affection and is more benign than the cytotoxic effects of expressed FHL1 protein associated with pathogenic missense variants.

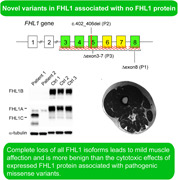

Emery‐Dreifuss muscular dystrophy (EDMD) is a rare muscle disease characterized by the clinical triade of early‐onset joint contractures, progressive muscle weakness, and cardiac involvement. EDMD is associated with pathogenic variants in five different genes: *EMD* (EDMD1), *LMNA* (EDMD2, EDMD3), *SYNE1* (EDMD4), S*YNE2* (EDMD5), and *FHL1* causing the subtype EDMD6. *FHL1* is located on the X‐chromosome and comprises eight exons that transcribe into three major isoforms; FHL1A, FHL1B, and FHL1C with distinct roles (Bonne et al., 2019). FHL1A is the predominant isoform and is highly expressed in the heart and skeletal muscle (Shathasivam et al., 2010). *FHL1* variants cause a wide spectrum of skeletal muscle and cardiac diseases, which can be divided into five main clinical presentations; hypertrophic cardiomyopathy (HC), reducing body myopathy (RBM), and X‐linked myopathy characterized by postural muscle atrophy (XMPMA), scapuloperoneal myopathy (SPM) and EDMD. In 2009, Gueneau et al. discovered *FHL1* as the causative gene of EDMD in seven families (Gueneau et al., 2009). Since then, 11 new *FHL1* variants have been reported to cause EDMD (Giucă et al., 2020; Gossios et al., 2013; Malfatti et al., 2013; Pen et al., 2015; San Román et al., 2016; Tiffin et al., 2013; Willis et al., 2016), almost all with a severe phenotype and all with some residual expression of FHL1 proteins. We report three families each with a novel variant in *FHL1*, suspected to result in complete loss of all three FHL1 isoforms.

Three unrelated probands were included in this study. Patient data are summarized in Table 1.

**Table 1:** Clinical, genetic, and age of of FHL1 patients

				Muscle weakness						
	*FHL1* variant	Age of onset/symptom	Current age	Upper limbs	Lower limbs	Axial	Facial	HCM/LVEF	Contractures	Other
Patient 1 (Danish)	NC_000023.11:g.(?_136209656)_(136211609_?)del (Δexon8)^a^	Early school age/dysphonia	†/24	N	Distal (MRC 4)	N	Y	Y/>60%	Elbows, ankles	Scoliosis
Patient 2 (Danish)	c.402_406del/p.Gln134Hisfs^a^9^a^	17/WPW	30	N	Proximal (MRC 4+)	Y (MRC 2)	N	Y/50%	Ankles	Dysphonia
Patient 3 (Austrian)	NC_000023.11:g.(?_136206005)_(136209540_?)del (Δexon3‐7)^a^	38/walking difficulties	58	Proximal (MRC 4), distal (MRC 4+)	Proximal (MRC 4), distal (MRC 4‐)	N	Y	Y/n.a.	Elbows, ankles	Dysphonia

Abbreviations: HCM, hypertrophic cardiomyopathy; LVEF, left ventricular ejection fraction; MRC, Medical Research Council; n.a., not available; N, no; WPW, Wolff–Parkinson–White; Y, yes.

^a^ NM_001449 and NC_000023.11 were used as reference sequences.

Patient 1: This patient presented with progressive dysphonia. On evaluation at age 18 years, he exhibited dysmorphic features with a long, slim face, high‐arched palate, atrophy of the masseter muscles, weakness of facial musculature, and ptosis. He had severe scoliosis, slight contractures across elbows, and tight Achilles tendons. In the lower extremities, he had slight atrophy of the right thigh and lower leg muscles and muscle weakness at ankle dorsiflexion. The patient died at age 24 years from acute heart failure due to thrombotic lung emboli from the pelvic veins. Patient 1 had mild hypertrophic cardiomyopathy with normal ejection fraction on echocardiography. Electrocardiography (ECG) was normal.

Patient 2: He was diagnosed with Wolff–Parkinson–White syndrome (WPW) at age 17 years, and subsequently with non‐obstructive hypertrophic cardiomyopathy. At age 30 years, he developed muscle pain, proximal muscle weakness, and mild dysphonia. Upon evaluation at age 30 years, he had tight Achilles tendons, discrete asymmetric ptosis, muscle weakness at truncal extension but no scoliosis, and hip flexion weakness. At age 31 years, implantation of an implantable cardioverter‐defibrillator (ICD) was performed after an episode with nonsustained ventricular tachycardia. Echocardiography showed a left ventricular ejection fraction of 50%, lateral apical and septal hypertrophy (maximum wall thickness 16 mm). ECG showed T wave inversion in the anterior chest wall. He is treated with Verapamil 120 mg twice a day.

Patient 3: This man developed progressive difficulties walking at age 38 years, requiring crutches from age 48 years. Upon evaluation, he had dysphonia, weakness of facial musculature, and ptosis. He had mild contractures of the elbow joints, tight Achilles tendons, and symmetrical weakness of the arms and legs. He had hypertrophic cardiomyopathy. ECG had signs of left ventricular hypertrophy.

In the mother of patient 1, echocardiography, ECG, and cardiac magnetic resonance imaging (MRI) were normal. The mother of patient 2 had mild hypertrophic cardiomyopathy (maximum septal wall thickness 12 mm) and ECG showed T wave inversion in the anterior chest wall, not requiring medical treatment. Pulmonary function was normal in all three patients and the two mothers, except for nocturnal orthopnoea and hypoventilation in patient 1.

MRI studies were performed using a 3.0 T Siemens scanner (MAGNETOM Verio Tim System; Siemens AG). T1‐weighed MRI in patient 2 revealed extensive fat replacement of erector spinae and milder involvement of hamstring muscles and anterior and lateral muscle compartments of the calves (Figure 1a). Patient 3 had a similar pattern of muscle affection. Milder affected leg muscles showed signs of edema on short tau inversion recovery (STIR) images, indicative of disease activity. T1‐weighted whole‐body muscle MRI of the two mothers was normal.

**Figure 1:**
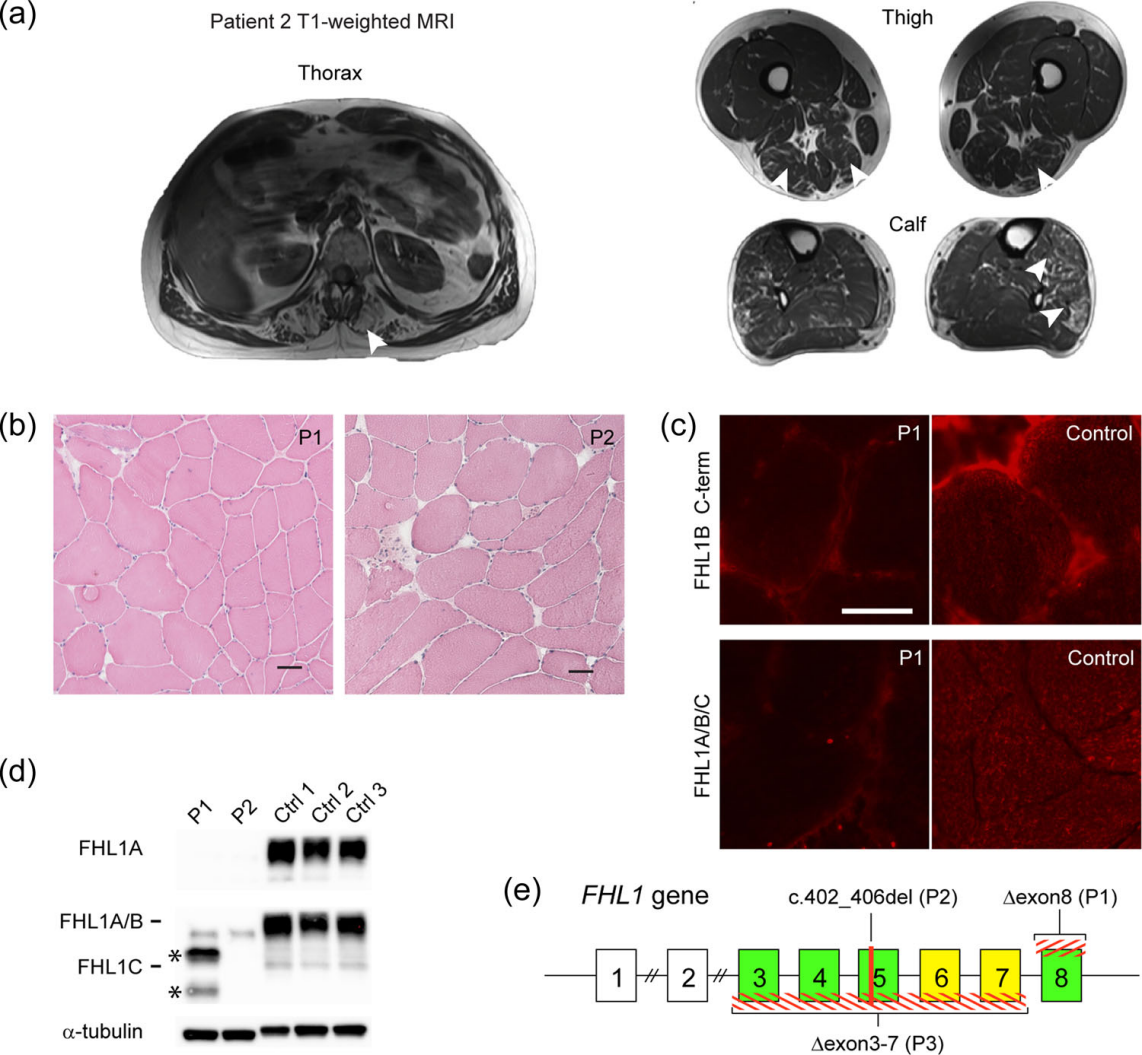
Muscle affection and FHL1 protein expression in FHL1 myopathy. (a) T1‐weighted MRI of patient 2 demonstrates the extensive replacement of muscle by fat in erector spinal muscles at thoracic levels (arrowheads). Moderate replacement of muscle by fat in the long head of the biceps femoris muscles, right semimembranosus muscle, tibialis anterior, and peroneal muscles (arrowheads). (b) Hematoxylin and eosin stain demonstrates moderate myopathy with internally nucleated fibers and variable fiber size in patients 1 and 2. (c) Immunostain for FHL1 using two different antibodies, one specific for only the FHL1A isoform, the other reacting against all three FHL1 isoforms, in patient 1, demonstrates absence of all normal FHL1 isoforms compared to healthy control (ctrl1, female age 72 years; ctrl2, male age 26 years; ctrl3, male age 45 years). (d) Western blot analysis using the same two antibodies as for immunostain shows no normal FHL1 protein expression of any isoform in patients 1 and 2, however, patient 1 may have modified FHL1A/C proteins (shown with asterisk). (e) The *FHL1* gene, where exons 1 and 2 are noncoding (white boxes) and exon 3–8 give rise to three alternatively spliced mRNA transcript (*FHL1A, exons 3–6, 8*; *FHL1B, exons 3‐8*; and *FHL1C, exons 3–5, 8*) by alternative splicing (yellow boxes are alternatively spliced exons, while green exons are translated in all transcripts). The variants of individual patients are shown in red (larger deletions are shown crosshatched). Bar = 50 µm.

Muscle tissue from vastus lateralis was obtained using a biopsy needle, flash‐frozen, cryosectioned, and stained with hematoxylin and eosin for general histopathology. Muscle biopsies from the three patients demonstrated mild to moderate myopathy with fiber size variation, sporadic necrosis, and few centrally nucleated fibers as a sign of ongoing degeneration‐regeneration cycles (Figure 1b). Immunohistochemistry and western blot analysis methods are described in detail in the Supporting Information Material. Immunohistochemical stains were made on cryosections from patient 1 using antibodies against the N‐terminus of FHL1 (1:200 dilution; Abcam) and FHL1B C‐terminal (1:100 dilution; Abcam). This staining demonstrated no protein expression (Figure 1c). Consistent with the immunostained sections, immunoblotting demonstrated complete lack of all three normal FHL1A/B/C transcripts in patients 1 and 2 (Figure 1d). However, in patient 1, bands were present that could be modified FHL1A and C transcript due to the introduction of an alternative termination codon as the shift in relative mobility from 32 to 26 kDa for FHL1A and 22 to 19 kDa for FHL1C corresponds to loss of amino acids encoded by exon 8.

Genetic analyses were initially performed by exome sequencing in a clinical setting (see Supporting Information Material). The breakpoints of the two deletions were subsequently mapped by standard techniques. NM_001449 and NC_000023.11 were used as reference sequences. Pathogenic variants are listed in Table 1. Patients 1 and 3 were hemizygous for deletion of exon 8 and exon 3–7, respectively in *FHL1*, which was identified after quantitative analysis of the WES data set. Patient 2 had a 5 bp deletion in exon 5 predicted to create an out‐of‐frame transcript (Figure 1e). The mothers of patients 1 and 2 were carriers, whereas segregation analysis of patient 3 was not performed. The novel variants are submitted to ClinVar for future reference. None of the three variants have to our knowledge, been described previously, and all absent gnomAD and human gene mutation database professional subscription.

We report three unrelated patients each with a novel pathogenic variant in *FHL1*. All three patients were in the mild phenotypical spectrum with late‐onset, slowly progressing muscle weakness. However, patient 2 had mild skeletal muscle involvement but a severe cardiac phenotype with early‐onset conduction defects, ultimately requiring implantation of an ICD. This is unusual, as muscle weakness typically precedes cardiac arrythmias and corresponds to the severity of muscle weakness (Madej‐Pilarczyk, 2018). Patient 1 died at age 24 from multiple lung embolies. There was no history of immobilization before the event and thrombocyte count was normal. Patients with mutations in FHL1 are not expected to have a higher risk of thromboembolic events than expected from the underlying risk of cardiac disease and the autopsy report did not suggest that EDMD6 was the cause of death. The reported variants are expected to affect all three transcripts of the three FHL1A, ‐B, and ‐C isoforms, which in patients 1 and 2 was supported by immunoblotting and immunohistochemical staining revealing the complete absence of all three isoforms. The same is predicted for patient 3 hemizygous for a large deletion. As for the potentially modified FHL1 proteins of patient 1, it is presently not possible to determine if they have any impact on the pathology. Cell studies have suggested that aberrant FHL1 protein exerts a cytotoxic function on muscle cell (Wilding et al., 2014). Such an effect is not observed in patients with predicted loss‐of‐function (LOF) variants, as our patients. Additionally, in the absence of FHL1A, an increase in FHL1C has been seen in several patients, which may affect the function of the potassium channel Kv1.5 and cause atrial fibrillation, a hallmark for patients with LOF variants (Gossios et al., 2013; Poparic et al., 2011; Tiffin et al., 2013). In agreement with this, our patients with no functional FHL1 protein or truncated for the 32 terminal amino acids of FHL1A, all displayed mild muscle involvement. Variants in the *FHL1* gene affecting the zinc‐coordinating cysteine and histidine residues in the second LIM domain causes RBM, while variants affecting the stability of the fourth LIM domain cause XMPMA. Variants that appear to affect the second LIM domain but not zinc‐binding may cause SPM. Rigid spine syndrome  has only been reported in one patient with a 9‐base deletion affecting the second LIM‐domain. Variants leading to amino acid changes in domains other than the second or truncated protein generally cause cardiomyopathy (HC) (Gueneau et al., 2009; Schessl et al., 2011). In some EDMD6 patients, an extended phenotype was observed including facial dysmorphology, pulmonary artery hypoplasia, genital abnormalities and sudden cardiac death. All these patients carried *FHL1* mutations leading to complete loss of FHL1A and FHL1B, while FHL1C was significantly overexpressed (Pen et al., 2015; Tiffin et al., 2013). One patient with a complete deletion of the *FHL1* and *MAP7D3* genes has been reported, leading to the absence of functional FHL1 as predicted in our probands. Although the phenotypic consequence can be difficult to interpret, when two genes are knocked out, the patient did have a mild phenotype presenting with muscle hypertrophy (Willis et al., 2016).

To date, the molecular basis of *FHL1‐related* myopathies remains unclear. In general, EDMD6 is associated with reduced protein levels (Gueneau et al., 2009). In the most severe *FHL1‐related* disorder, RBM, FHL1 remains at wild type levels, and muscle histopathology reveals intracytoplasmic inclusion bodies that stain positive for numerous proteins, including FHL1 (Wilding et al., 2014). Together, this supports the hypothesis that complete loss of all FHL1 isoforms result in a milder phenotype than the cytotoxic effects of expressed FHL1 transcripts with pathogenic missense variants, which so far is the prevailing hypothesis for pathogenesis of EDMD (Wilding et al., 2014). Thereby, our study gives further insights into the pathogenesis and genotype‐phenotype relation of FHL1‐related diseases.

In this study, the mothers of the two patients are asymptomatic, but cardiac examination revealed mild hypertrophic cardiomyopathy in one. The phenotype of female EDMD6 carriers has only been investigated in six case reports of EDMD6 (Gossios et al., 2013; Gueneau et al., 2009; Knoblauch et al., 2010; Pen et al., 2015; San Román et al., 2016; Tiffin et al., 2013). Previous findings include rigid spine and thickened left ventricular wall (Knoblauch et al., 2010; San Román et al., 2016; Tiffin et al., 2013).

In conclusion, our findings support the theory that negative effects from cytotoxic aberrant FHL1‐protein produce a more severe phenotype compared to complete loss or in‐frame truncation of FHL1 as found in the presented patients (Gueneau et al., 2009; Willis et al., 2016).

## CONFLICT OF INTEREST

The authors declare no conflict of interest.

## Supporting information

Supporting information.
