# Supplementary material for: Three novel FHL1 variants cause a mild phenotype of Emery‐Dreifuss muscular dystrophy
Source: Hum Mutat. 2022 Jul 16;43(9):1234–8. doi: 10.1002/humu.24415 (PMC9545859; doi:10.1002/humu.24415)
Supplement: Supplementary file 1 — Supporting information. [file HUMU-43-1234-s001.pdf]

Three novel *FHL1* Variants cause a mild Phenotype of Emery-Dreifuss Muscular Dystrophy

Supplementary material

Methods

Immunohistochemical staining

Vastus lateralis biopsies were sectioned at a thickness of 10µm on a cryostat (NX70, Thermo Fisher Scientific, Waltham, MA). Sections were fixed in ice-cold acetone and subsequently blocked in buffer (3% fetal calf serum in PBS) prior to staining. For assessment of FHL1 expression two antibodies were used, one specific for the penultimate C-terminal of FHL1A at 1:100 in blocking buffer (ab23XXX, Abcam, Cambridge, UK), the other specific for the N-terminal of FHL1A/B/C at 1:200 in blocking buffer(ab 59XXX, Abcam). Sections were incubated in primary antibody over night at 4 degrees followed by incubation with Alexa Fluor 594 secondary antibody for 3 hours at room temperature. All sections were observed at room temperature using a Nikon 20x Plan Apo VC N/A 0.75 mounted on a Nikon Ti-E epi-fluorescence microscope (Nikon Instruments, Melville, NY). Images of the entire sections were acquired at 20x with a 5Mpixel Andor Neo cameras for fluorescence imaging (Andor, Belfast, Northern Ireland) using NIS-Elements Advanced Research (BR/AR) software (Nikon). All bars in micrographs are 50 µm.

Western blotting

Vastus lateralis muscle biopsies were sectioned on a cryostat and homogenized in ice-cold lysis buffer with protease and phosphatase inhibitors (10 mM Tris, pH7.4, 0.1% Triton-X 100, 0.5% sodium deoxycholate, 0.07 U/ml aprotinin, 20 µM leupeptin, 20 µM pepstatin, 1 mM phenylmethanesulfonyl fluoride (PMSF), 1 mM EDTA, 1 mM EGTA, 1 mM Dithiothreitol, 5 mM β-glycerophosphate, 1 mM sodium fluoride, 1.15 mM sodium molybdate, 2 mM sodium

pyrophosphate decahydrate, 1 mM sodium orthovanadate, 4 mM sodium tartrate, 2 mM imidazole, 10 nM calyculin, 5 mM cantharidin, Sigma-Aldrich, St. Louis, MO) using a Bullet Blender bead-mill at 4°C (Next Advance Inc., Averill, NY). To investigate the FHL1 expression in patients and controls equal amounts of extracted muscle proteins (3 µg/µl) were separated on 4-15% or 7% TGX polyacrylamide gels (Bio-Rad, Hercules, CA) at 200 V for 30 minutes. Proteins were transferred to polyvinyldifluoride (PVDF) membranes at 2.5 A for 5 minutes using a Trans-Blot Turbo (Bio-Rad) and blocked in Bailey's Irish Cream (Dublin, Ireland) for 30 min and washed in Tris-buffered saline with 1% tween-20 (TBS-T) to remove excess Bailey's. Membranes were incubated over night at 4°C with the same primary antibodies against FHL1 as used for immunohistochemistry. As a loading control, anti-α-tubulin was used at 1:100,000 (clone 12G10-c [270µg/ml], DSHB). Secondary goat anti-rabbit and goat anti-mouse antibodies coupled with horseradish peroxidase diluted at 1:10000 were used to detect primary antibodies (DAKO, Denmark). Immuno-reactive bands were detected using Clarity (Bio-Rad, Hercules, CA) and visualized using a ChemiDoc digital darkroom (Bio-Rad).

Clinical genetics

Patient 1 and 3 was identified through the MyoSeq study (Nat Rev Neurol. 2016 May;12(5):294-309. doi: 10.1038/nrneurol.2016.35) whereas patient 2 was identified by in-house whole-exome sequencing (WES) in a clinical setting using Human Core Exome Kit capture (Twist Bioscience, South San Francisco, CA) followed by sequencing (100bp paired-end) on a NovaSeq 6000 (Illumina, San Diego, CA) to a mean depth >80x (98%, >20x). Data were processed according to best practice guideline for GATK 3.8 and variant analysis was performed using VarSeq (Golden Helix, Bozeman, MT). All procedures were essential carried out according to the descriptions of the manufacturers.
